# Review: Modes and Processes of Severe Plastic Deformation (SPD)

**DOI:** 10.3390/ma11071175

**Published:** 2018-07-10

**Authors:** Vladimir Segal

**Affiliations:** Engineered Performance Materials, 2874 Laurel Ridge Ln, Howell, MI 48843, USA; vladimirsegal@comcast.net

**Keywords:** severe plastic deformation (SPD), mode deformation, simple shear and pure shear, structure modification, SPD techniques

## Abstract

In this review, severe plastic deformation (SPD) is considered as a materials processing technology. The deformation mode is the principal characteristic differentiating SPD techniques from common forming operations. For large plastic strains, deformation mode depends on the distribution of strain rates between continuum slip lines and can be varied from pure shear to simple shear. A scalar, invariant, and dimensionless coefficient of deformation mode is introduced as a normalized speed of rigid rotation. On this basis, simple shear provides the optimal mode for structure modification and grain refinement, whereas pure shear is “ideal” for forming operations. Special experiments and SPD practice confirm this conclusion. Various techniques of SPD are classified and described in accordance with simple shear realization or approximation. It is shown that correct analyses of the processing mechanics and technological parameters are essential for the comparison of SPD techniques and the development of effective industrial technologies.

## 1. Introduction

The application of large, intensive, and even severe strains is not a new concept in metalworking. It has been practically used since ancient times. With ordinary forming operations, such as forging, rolling, drawing, and so forth, sufficiently large strains can be attained at hot, warm, and cold temperatures. D. Kulhman-Wilsdorf [1] developed the general framework for the evolution of dislocation structures at moderate strains. N. Hansen [2] extended this framework to large strains that are present during rolling and drawing. In 1930–1950th, P.W. Bridgman [3] introduced a new technique of unlimited straining by torsion under compression (or high-pressure torsion, HPT) and performed pioneering works on the processing of various materials. He first described the dramatic refinement of grain structures induced by very large shear. Later, this was confirmed and extended further, and new techniques for transforming simple shear into potential industrial operations were suggested. These results stimulated intense research on the use of severe plastic deformation (SPD) for prospective application in materials science and processing. A short review of the history of SPD will be presented in the following section. In the field of material science, numerous publications on SPD have dealt with structures and structure-property relations. They have been reviewed by many authors and are not considered here; for information, readers are referred to [4,5,6]. In the scope of the present review, it is important to note that the effects of SPD, not observed for ordinary forming operations, were discovered through the development of new processing techniques. Published data demonstrate that structure evolution under identical conditions strongly depends on the processing mechanics. These mechanics distinguish SPD from ordinary forming operations, as a property-driven processing technology, providing unique material modification.

However, very few works have presented in-depth analysis of the SPD mechanics. Known publications usually use approximate methods, sometimes with inadequate formulations, which do not capture the specifics of continuum stress-strain states. Although the dissimilarity of SPD techniques to ordinary operations is well recognized and is often mentioned in the literature, there is not a clear understanding of their principal differences or a definition of SPD itself. According to [7], SPD is “any method of metal forming under *extensive* hydrostatic pressure that may be used to impose a *very high* strain on a bulk solid without introduction of any *significant* change in the overall dimensions of the sample and having the ability to produce *exceptional* grain refinement” (the italics are the author’s). As will be seen later, this description contains secondary and indeterminate characteristics of individual SPD techniques and cannot be used as the universal definition. It does not correlate the processing mechanics and mechanisms of structure evolution, which is essential for the development of optimal techniques and industrial SPD technologies.

Since Bridgeman’s works, processing techniques have played a critical role in SPD. In the beginning, a few techniques were introduced in which simple shear was the dominant deformation mode, similar to torsion under compression. It was accepted in the field that SPD is intrinsically associated with shear deformation. During further development, several new methods were also suggested. Many of them presented various combinations or alternative designs of the basic ideas; however, there were also examples of innovative approaches. Currently, the total number of known SPD techniques exceeds 60 and the development of new methods may be expected in the future. For some of them, deformation mode only partly approximates or just imitates simple shear. In other cases, simple shear is not evident or is hidden under inhomogeneous and complex stress-strain states. In some instances, ordinary forming operations were successfully used as SPD techniques. Finally, a few recent publications have claimed that pure shear is also effective for SPD processing. The aforementioned controversial results should be carefully analyzed and reviewed to compare the efficacy of various techniques and to develop a sound basis for the formulation of the optimal mode(s) for structure modification.

Presently, various SPD techniques are known for bulk batch and continuous billets; for rods, plates, tubes, and sheet materials; for one-step and multi-step processing; and for SPD of surfaces and thin cross-sections. However, there are strong limitations in terms of their applicability to industrial products, especially those with a complex shape and large size. Research and development on SPD are usually performed on a laboratory scale using small samples, the simplest tools, and labor-intensive procedures, which do not meet practical requirements. Some laboratory operations are not suitable for industry. Regardless of the preferable mode of deformation, each processing technique has specific mechanical parameters such as stress, strain, strain rate, stress and strain distributions, contact friction and pressure, load, energy, and so forth. Additional important characteristics are product cost, process simplicity, reliability, and, especially, potential to scale up. As most of this information remains unknown, significant efforts are needed for the engineering development and commercialization of SPD. To reduce risk and expenses, various SPD techniques should be reviewed and reevaluated for industrial applications.

The present review is intended to fulfill some of these aims. First, a definition of the deformation mode is introduced as a normalized and dimensionless strain rate spin. All possible modes range between simple shear and pure shear. The structural effects of these limit cases are discussed with an emphasis on the role of processing mechanics. It is shown that approximation to simple shear is the basic characteristic of SPD. Practical options for technical realization of the simple shear are considered. Finally, the most popular techniques are compared from the practical point of view.

## 2. On the History of SPD

The origin of SPD is traced to the work of Bridgman [8]. In this paper, he described structure refinement after intensive HPT to the extreme grain size now identified as “nanograin size”. Bridgeman performed extensive research on HPT of different materials and made numerous discoveries; however, for some reason, only refinement was identified as the subject and goal of SPD. Subsequent progress in SPD research in the areas of materials science and processing technology was delayed and uneven, and the real history is still being written. Nevertheless, a few assays were devoted to the history of SPD [9,10], and the post Bridgeman development of HPT was overlooked in [11]. These recent papers did not include seminal works published in the 1970s and 1980s, particularly in Russian. Therefore, they do not provide a complete picture. A review [6] presented a more accurate history of SPD. Therefore, additional discussion is necessary using a chronology of the original works on SPD as the only reliable historical background.

In the period from 1950 to the 1970s, HPT was first applied to organic and non-organic materials in quantum-mechanics chemistry. That resulted in the discovery of new compounds and unusual reactions, stimulating similar research on metals. Regarding grain refinement, Bridgman [8] conducted X-ray analysis, which was the only available methodology for the investigation of such fine structures at the time. J. Nutting [12] first applied a TEM analysis to heavily HPT-processed samples and presented direct observations of the attainable microstructures. He described them as sub-micron, near-equiaxed, and dislocation-free grains with sharp high-angle boundaries. This description conforms to the current definition of ultrafine-grained (UFG) microstructures. It was later confirmed by S. Erbel [13], A. Korbel et al. [14], I. Sanders and J. Nutting [15], Rigney et al. [16], and N.A. Smirnova et al. [17]. By the mid-1980’s, structure refinement during HPT to the submicron grain size was authentically proved by the modern analytical methods. In fact, this marks the beginning of the so-called “microstructural age” of SPD. In the period from 1980 to 1990, R.I. Kuznetsov et al. [18] started a vast program on SPD at the Institute of Metal Physics, Sverdlovsk, USSR, where the HPT technique and microstructural analysis were applied to various problems: the strain hardening of steels [19], processing and recrystallization of single crystals [20], phase transformation in iron-nickel alloys [21], and others. As a part of this program, a similar work was carried out on the superplasticity of HPT-processed Al alloys [22]. However, HPT could only produce very small samples, which did not present a practical interest, and SPD remained an area of limited academic activity.

The situation changed after the invention of a new processing technique in 1972 known as equal-channel angular extrusion (ECAE) or equal-channel angular pressing (ECAP) [23,24]. ECAE transformed SPD into a simple forming operation that could be applied on an industrial scale to large billets from various materials under various processing conditions. Extensive research and development on ECAE was performed at the Institute of Technical Physics (FTI), Minsk, USSR from 1970 to 1986. For the first time, bulk samples with ultrafine-grained structures were prepared and their mechanical and physical properties were investigated. In particular, the significant increase of yield stress and ultimate tensile strength with the preservation of sufficiently good ductility for highly hardening conditions, additional strengthening by following deformation processing, and property improvement of the special elastic, superconductive, and magnetic materials were demonstrated [25,26]. ECAE was effectively applied to the breakdown of cast ingots, consolidation and bonding of powders, and fabrication of laminates and composites. In fact, this activity appears to be the actual turning point that transformed SPD into a material processing technology and triggered intensive research in the field. Y. Estrin and A. Vinogradov [6] clearly stated that the SPD revival is due to work performed in Minsk in the 1970s and 1980s.

It is necessary to underline again that new deformation techniques led to two landmarks in the history of SPD: in the first case, HPT established SPD as a scientific concept, and, in the second case, ECAE transformed SPD into a materials processing technology.

## 3. Modes of SPD

The term “deformation mode” is usually used in conjunction with particular methods of material processing or testing without characterization of their specifics. A more adequate definition of deformation mode based on properties of stress, strain, and strain rate tensors was introduced in [27]. For strains ε >> 1, which are typical during SPD, the effects of elasticity and hardening can be neglected, and the material behavior approximates the ideal rigid-plastic body. Also, for simplicity, plane (two-dimensional) and uniform plastic flows will be considered below. Following R. Hill [28], large deformation of such material is described by the flow theory of Levy-Mises using the stress tensor **T_σ_** and strain rate tensor **T_ξ_** in the directions of the principal shear stresses, known as the α- and β-slip lines. Since plastic flow is independent of hydrostatic pressure, all stress tensors normalized by the material yield stress are identical.

**Figure 1:**
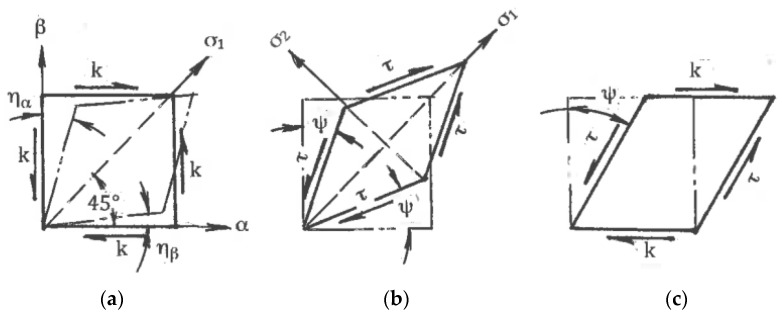
The element distortion along slip lines: (**a**) general case; (**b**) pure shear; (**c**) simple shear.

Associated strain rates are shown in Figure 1, where η_α_ and η_β_ are the shear rates along the slip lines. To compare different strain rate states, tensors **T_ξ_** should be normalized by the effective von Mises shear strain rate:

η = (η_α_ + η_β_)/2 (1)

Here, η_α_ = du/d_β_, η_β_ = dv/d_α_, and u and v are velocity components along the _α_- and _β_-slip lines, respectively. The normalized tensors **T_ξ_**/η vary depending on the distribution of shears between the slip lines. This can be evaluated by the coefficient of deformation mode:

C = (η_α_ − η_β_)/2η (2)

The coefficient C is scalar and dimensionless. In one limit case, shear strain rates are uniformly distributed between both slip lines: η_α_ = η_β_ and C = 0. This case corresponds to pure shear (Figure 1b). In another limit case, the shear strain rate is localized along the α-slip line with η_α_ = 2η and C = 1 or along the β-slip line with η_β_ = 2η and C = −1. This case corresponds to simple shear along either the α- or β-slip lines (Figure 1c). Because the two slip lines are equivalent, the coefficient C is changed in the range of −1 ≤ C ≤ 1, and all possible deformation modes are confined between pure shear and simple shear. According to (1) and (2), the normalized strain rate tensor can be resolved into two parts:

**T_ξ_/**η = {0, 0, 0, 1, 1, 0} + {0, 0, 0, C, −C, 0} (3)

The first term in (3) corresponds to the normalized pure shear, whereas the second term defines the normalized speed of rigid rotation. Parameters C and η fully describe the linear strain rates ξ_α_, ξ_β_, the shear strain rates η_α_, η_β_, and the angle speed ω of rigid rotation along the slip lines:

ξ_α_ = ξ_β_ = 0, η_α_ = η(1 + C), η_β_ = η(1 − C), ω = ηC, (4)

Equation (4) show that C is the normalized speed of rigid rotation C = ω/η. Similar strain rates in the directions of arbitrary coordinate axes x and y are:

ξ_xx_ = −ξ_yy_ = ηsin2ϕ, η_xy_ = η(cos2ϕ + C), η_yx_ = η(cos2ϕ − C), ω = ηC (5)

**Figure 2:**
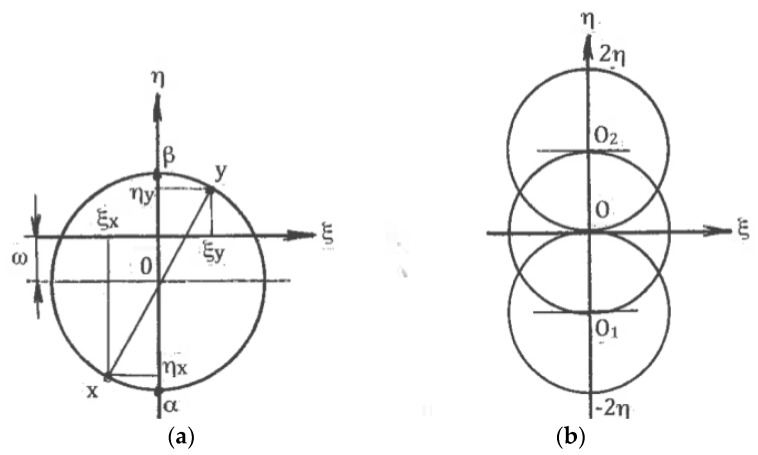
Moore’s circles with rotation (**a**) and in the limit cases of pure shear and simple shear (**b**).

Here, ϕ is an angle between the x-axis and the α-slip line in the counter-clockwise direction. In the classic theory of plasticity, the rigid rotation is usually neglected. However, in the materials with a structure, it plays an important role. A geometrical representation of formulae (3) to (5) by the Moore circle is shown in Figure 2a. Figure 2b outlines Moore circles for all possible deformation modes between pure shear (center O), simple shear along the α-slip line (center O_1_), and simple shear along the β-slip line (center O_2_).

## 4. Simple Shear Versus Pure Shear

Plastic deformation changes the shape, structure, and properties of a material. The structural evolution presents the main focus of interest in SPD, and it shows a strong influence of deformation mode. It is informative to compare the specific effects of deformation mode for the limit cases of simple shear and pure shear using available analytic and experimental results.

### 4.1. Finite Strains

The effects of SPD are detected after finite strains. Distortions of material elements by pure and simple shear during uniform steady flows with the same effective shear strain rate η (1) show [29] that for pure shear, square elements are transformed into rhombs oriented along fixed directions of the principal stresses σ_1_ and σ_2_ (Figure 1b), whereas for simple shear, the same square elements are transformed into parallelograms rotating continuously to the directions of the α- or β-slip lines (Figure 1c). These parallelogram and rhomb configurations cannot be interchanged by any rigid rotation. However, round elements in both cases are transformed into ellipses, allowing a formal geometrical comparison of distortions after finite pure shear and simple shear. In both cases, dissipation of the plastic work within a “unit” material element during time t is:

A = 2ηkt = γk(6)

Here, γ = 2ηt is the effective von Mises shear strain. Equations (3) and (6) demonstrate that the plastic work is independent of C, and for the same effective strain rate (η = const), different modes are energetically equivalent. For pure shear, the principal strains in fixed directions are:

ε_1_ = −ε_2_ = γ/2 (7)

For simple shear, linear strains along the ellipse axes are related to γ by:

ε_1_ = −ε_2_ = ln{[2 + γ^2^ + γ(γ^2^ + 4)^1/2^]/2}/2 (8)

Orientations of strains ε_1_ and ε_2_ coincide with principal stresses σ_1_ and σ_2_, and are the effective von Mises strains only for pure shear. In the case of simple shear, strains ε_1_ and ε_2_ from Equation (8) rotate relative to σ_1_ and σ_2_ and cannot be used as the effective strains. Distortions in both cases show a noticeable difference with the increase of strains. For the same energy (6), pure shear leads to the maximum geometrical distortion [29]. Hill [30] used this idea and introduced “ideal forming” operations in which pure shear takes place along any streamline of a steady plastic flow. “Ideal forming” provides zero abundant work, the lowest load, and a uniform strain distribution across the processed material. Practically, it is hard to attain “ideal forming” in industrial operations due to contact friction and specific tool configurations. Richmond and Devenpeck [31] introduced special “sigmoidal dies” for “ideal forming” during frictionless drawing and extrusion. Sufficiently good approximation also provides slowly convergent flows for multi-pass rolling and drawing with small reductions per pass and low friction when |C|→0.

### 4.2. Rigid Rotation

In materials processing for properties, it is not easy to formulate a simple and universal optimization principle such as “ideal forming” operations because of the variety of materials, deformation conditions, and operational mechanisms. In terms of the processing mechanics, the main continuum parameters are hydrostatic pressure, strain, strain rate, and deformation mode. While the first three parameters have been well investigated, the role of the deformation mode is not fully recognized. Deformation mode prescribes continuum strain rates and strains as boundary conditions for aggregates of grains outlined by slip lines. At the meso- and micro-scales, these conditions define crystallographic glides, accumulated shears, rotations, and the evolution of dislocations in the individual grains. Thus, deformation mode affects structural modification during processing. According to (3), a strain rate tensor at any location is reduced to pure shear and rigid rotation. For a homogeneous continuum, a uniform distribution of local rotations is equivalent to the rigid rotation of the whole body, which does not affect the mechanical state. For non-uniform distributions, variations of rotations can be neglected if their gradients are small. In these cases, continuum mechanics does not consider rotations and all deformation modes are reduced to pure shear. However, in materials with grain structures having different crystallographic orientations and glide systems, gradients of rotation between adjacent grains are large and should be taken into account. Correspondingly, deformation mode has a significant effect on the evolution of microstructure, texture, and properties. In heavily deformed metals, the original grains are subdivided for fragments of different orientations separated by high-angle, middle-angle, or low-angle boundaries [2]. Crystallographic glide within fragments of volume w_i_ corresponds to the local speeds of rotations ω_i_ and local deformation modes c_i_ = ω_i_/η. Toth et al. [32] suggested that the rotations of adjacent fragments continuously increase the lattice curvature along their boundaries, which eventually leads to the formation of new high-angle boundaries and fine grains. Selection of glide systems is described by the Taylor’s principle minimization of the plastic work in fragment agglomerates under boundary conditions prescribed by the continuum stress and strain rate tensors. The latter means that the average rotation rate or deformation mode of all fragments “i” should be equal to the continuum rotation rate ω or continuum deformation mode C of the whole volume W (for simple shear of strongly inhomogeneous materials, rigid rotation of microstructural elements is also possible):

Σω_i_w_i_ = ωW or Σc_i_w_i_ = CW (9)

It is obvious that local rotations and structure refinement are more effective for simple shear where |C| = 1 and |ω| = η than for pure shear where C = 0 and ω = 0. Gu et al. [33] performed crystal plasticity modeling for copper subjected to two passes ECAE and rolling with equivalent effective strains at room temperature. Computed and experimental results confirmed that the average grain rotation and refinement were much more intensive for simple shear during ECAE than for pure shear during rolling. Mishra et al. [34] and Kang et al. [35] obtained similar results. Such a mechanism of micro-rotations supposes a broad definition of “fragment”, irrespective of angles of misorientation, including grains, grain-subdivided areas, subgrains, cell blocks, and cells, as well as the continuous evolution of low-angle boundaries to high-angle boundaries during straining. Therefore, the maximum rotation is the same principal characteristic of simple shear, as the maximum distortion is the principal characteristic of pure shear.

### 4.3. Textures

**Figure 3:**
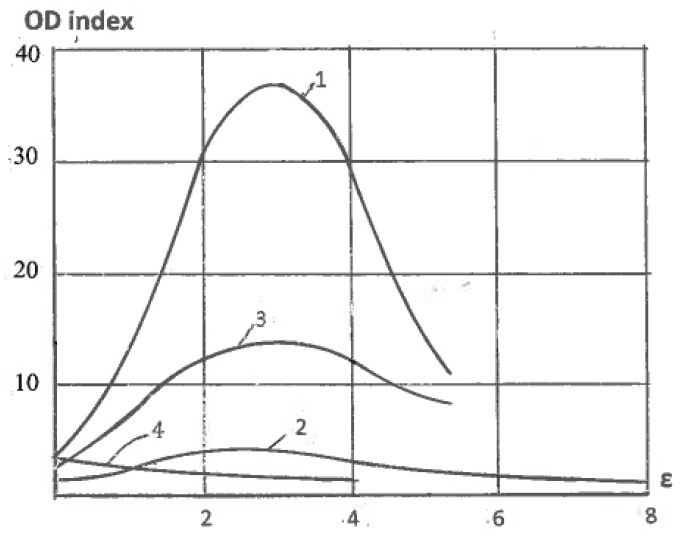
Texture strength (OD index) of Al0.5%Cu alloy versus effective strains after: 1—rolling of the original material; 2—ECAE of the original material; 3—rolling of the UFG material; 4—ECAE of the UFG material.

Another significant difference is the selection of glide systems within grains and fragments under simple shear and pure shear. They are also assigned according to continuum boundary conditions for grain aggregates: shear rate 2η along one family of slip lines is selected for simple shear (Figure 1c) and shear rates η along both families of slip lines are selected for pure shear (Figure 1b). Hirsch et al. [36] noted that textural analysis is very relevant to the correlation between structures and processing mechanics because deformation textures reflect collective crystallographic glides. For f.c.c. and b.c.c. metals with a sufficiently large number of slip systems, monotonic alignment of crystallographic glides to one direction of macroscopic slip lines takes place much faster than alignment to two directions of slip lines. Therefore, simple shear requires lower textural arrangement to the end orientations than pure shear. In fact, Huang [37] and S. Li and H. Li [38] observed the close alignment of active systems of crystallographic glide to a macro shear plane in dilute aluminum alloys, even after one ECAE pass with an effective strain of ε ~ 1. Comparison of the final texture orientations in different cases is difficult because of differences between the original textures, stresses/strains stated, and loading histories. However, important information allows a comparison of the texture strength induced by simple shear and pure shear. Ferrasse et al. [39] performed experiments using dilute aluminum alloy Al0.5%Cu subjected to simple shear by multi-pass ECAE and to pure shear by intensive rolling at room temperature up to effective strains of ε = 4.6. Texture strength was estimated according to the OD index as the root mean square of the peak values of ODF. Because the original material had a random-to-weak texture with OD = 2.2, the change in the texture strength was mostly induced by processing. The results shown in Figure 3 confirm that pure shear (curve 1) provides a very strong texture with the maximum OD = 37 times of random at ε = 2.3.

However, simple shear textures (curve 2) remain weak with the maximum OD = 37 times of random at the same strain of ε = 2.3, which is an order of magnitude lower than for pure shear. In both cases, a subsequent drop in the texture strength is attributed to structure refinement. After four passes of ECAE, the average grain size was refined from about 20 microns to 0.5 microns. Similar processing with multi-pass ECAE and rolling was performed on the obtained ultrafine-grained (UFG) material. The results are also presented in Figure 3 for pure shear (curve 3) and simple shear (curve 4). In both cases, the texture strengths of the UFG material were lower than those of its coarse-grained counterparts, but it remained much stronger for pure shear than for simple shear. Kang et al. (2007) also observed weakening of the texture strength and enhanced structure refinement due to shear deformation.

### 4.4. Hardening

**Figure 4:**
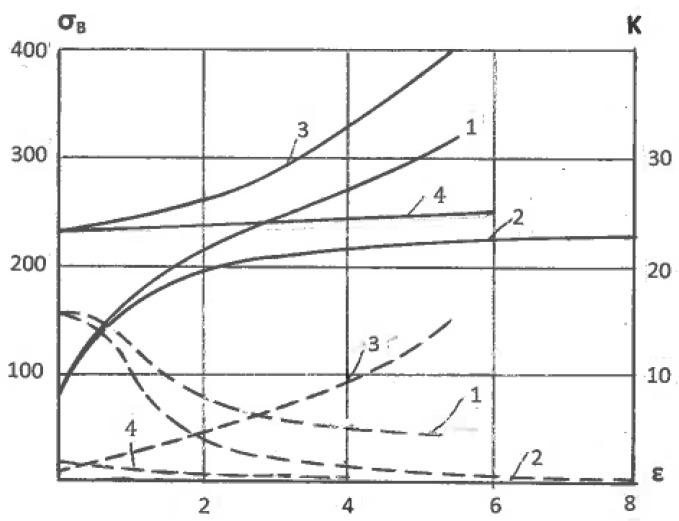
Ultimate tensile strength (σ_B_) (solid lines) and hardening coefficient K (dashed lines) of Al0.5%Cu alloy versus effective strains ε after: 1—rolling of the original material; 2—ECAE of the original material; 3—rolling of the UFG material; 4-ECAE of the UFG material.

The tensile strength of single-phase alloys processed by severe plastic deformation depends on dislocation, textural, and Hall-Petch hardening. Segal [40] investigated the effect of strains ε on the ultimate tensile strength σ_B_ of Al0.5%Cu alloy after rolling (curve 1) and ECAE (curve 2) (Figure 4). For ε < 2, σ_B_ is identical for both deformation modes, but it shows increased divergence with further straining. The stronger dislocation and textural hardening during pure shear suppress the prevalent hardening for structure refinement during simple shear. The same effect is observed after similar processing of the UFG material prepared by four passes of ECAE. A hardening diagram of the UFG Al0.5%Cu alloy after additional simple shear straining (Figure 4, curve 4) is a continuation of the original curve 2. However, when additional rolling changes the deformation mode during the preparation of the UFG material from simple shear to pure shear, the hardening diagram shows a continuous increase with increasing strain (Figure 4, curve 3). This difference between coarse-grained and ultrafine-grained materials is clearly demonstrated by the corresponding curves of 1–4 (Figure 4, dashed lines) for the hardening coefficient K = dσ_B_/dε. In the Al0.5%Cu alloy with strains of ε < 2, intergranular crystallographic glide is the dominant deformation mechanism for both pure shear and simple shear deformation modes. For pure shear, this mechanism continues to operate during further straining with continuously increased hardening. In the case of simple shear, the deformation mechanism of the UFG material is changed to grain boundary dislocation activity with insignificant hardening or even softening. However, when the same UFG material was subjected to pure shear by additional rolling, crystallographic glide within grains was restored, and hardening noticeably increased again. Similar findings were obtained in many studies. For example, Angella et al. [41] reported significantly lower hardening and strength after ECAE than after rolling with equivalent strains. Angella et al. [41] and Bahadori et al. [42] also observed that additional rolling transformed ultrafine equiaxed grains with sharp boundaries, induced by ECAE, into thin lamellar structures with strongly developed substructures and deteriorated ductility.

### 4.5. Localization

**Figure 5:**
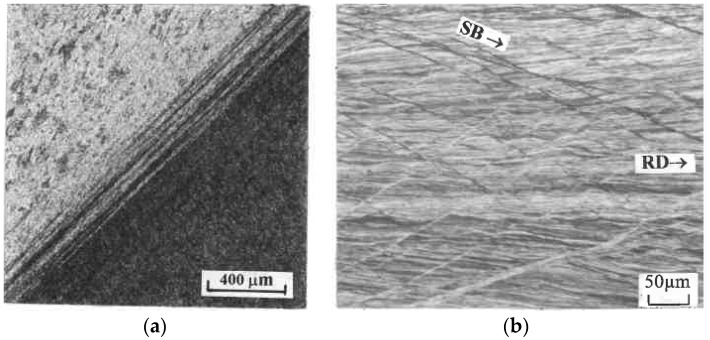
Shear band localization during ECAE (**a**) and rolling (**b**) of Al0.5%Cu alloy (SB-shear bands; RD-rolling direction).

If straining proceeds, continuous plastic flow is substituted by localization in micro- and macro-shear bands (SBs). Transition to localization takes place locally or globally when material hardening becomes sufficiently low or negative (softening). Related processes were considered at a macro-scale for pure shear and simple shear in [27,29,32]. Micro-shear bands first appear at certain crystallographic planes and, then, along the directions of slip lines. They grow, join into clusters, and form macro shear bands propagating through the whole material. As the deformation mode within shear bands conforms to simple shear, simple shear processing in conjunction with low hardening is the most favorable mode for early localization in contrast to pure shear processing providing strong hardening and delayed localization. Mishra et al. [34] and Sus-Ruzkowska et al. [43] considered ECAE as processing by localization. Examples of simple shear and pure shear localization are shown in Figure 5 for ECAE (a) and rolling (b), respectively. In the first case, localization displays a global character throughout the plastic zone, whereas it covers a small area and has an insignificant effect in the second case.

**Figure 6:**
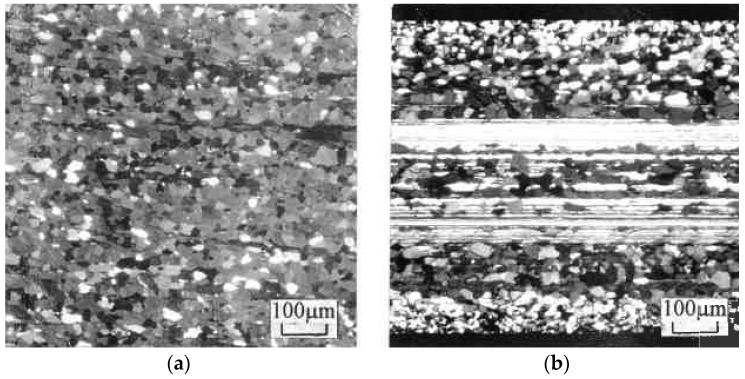
Microstructures of pure Al (99.9992%) after dynamic recrystallization induced by ECAE (**a**) and rolling (**b**) with effective strain of ε = 4.6.

**Figure 7:**
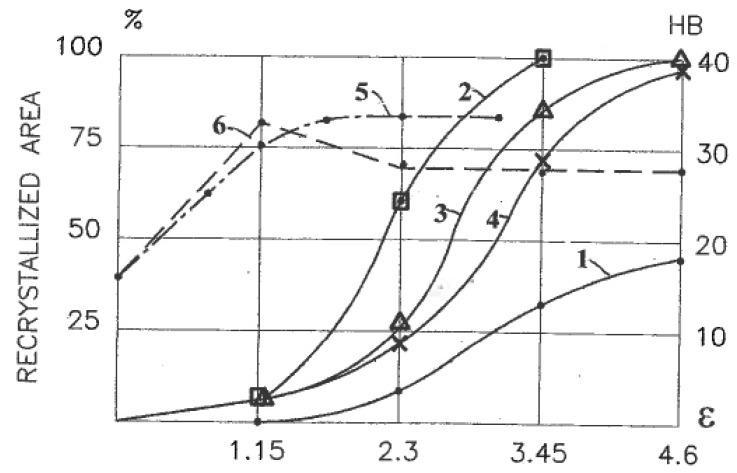
Dynamically recrystallized area (%) (solid lines) and hardness HB (dashed lines) of pure Al (99.9992%) versus effective strains ε: 1—rolling; 2—ECAE, Route D (B_C_); 3—ECAE, Route A; 4—ECAE, Route C; 5—rolling; 6—ECAE.

After localization starts, it accommodates the main part of the plastic work within shear bands. Because of high concentrations of stored energy, shear bands and their intersections influence structural evolution depending on the deformation mode. This conclusion was confirmed by experiments on the dynamic recrystallization of high purity aluminum Al5N2 (999,992%) [27]. Intensive cold deformation of this material results in the formation of grain nuclei at shear bands, which after recrystallization can be observed by optical microscopy. Related structures are presented in Figure 6 after four passes of ECAE (a) and after rolling reduction of 99% (b) at the same von Mises strain of ε = 4.6. For uniform simple shear during ECAE, the structure is fully recrystallized. For rolling, the picture is more complicated. In the middle of the cross-section with near-pure-shear deformation mode, recrystallization only takes place in a few deformation bands with specific crystallographic orientations, whereas it is not observed in most of the deformation bands. At the same time, a fully recrystallized very fine microstructure is detected in surface layers with intensive simple shear induced by dry contact friction. The quantitative results of the percentage of recrystallized areas (Figure 7) also demonstrate a significant difference between rolling (curve 1) and various loading paths of ECAE known as routes D (or B_C_) (curve 2), A (curve 3), and C (curve 4). Hardness HB of this soft material shows an increase to a stable amount for rolling (dashed curve 5) and evident softening after two passes of ECAE with ε = 2.3 (dashed curve 6).

### 4.6. Loading Paths

To accumulate very large strains, stimulate localization, and control the morphology of shear bands and their intersections, as well as global material distortion, SPD is usually performed in a few steps with a variety of loading paths. Such operations are complicated for pure shear (Figure 1b), which changes dimensions in two principal directions and leads to significant reduction of the material thickness. In contrast, simple shear (Figure 1c) preserves one of these dimensions, providing options for modification of the billet orientation between successive steps. Thus, intensive straining can be achieved in bulk materials.

### 4.7. Structure Refinement

Grain refinement during SPD has roused the biggest interest and numerous publications. SPD is a continuation of ordinary straining at ambient temperatures comprising the origination and interaction of dislocations and other defects, which form low-angle substructures of cells and sub-grains. Subsequent structure evolution during SPD includes fragmentation by grain splitting and the formation of cellblocks, micro-shear bands, and twins with further transformation of low-angle boundaries into high-angle boundaries due to continuous and discontinuous dynamic recrystallization. Related mechanisms are still disputable; however, the effects of rigid rotation, texture evolution, hardening, shear band localization, and their intersections due to the changing of loading paths are generally recognized. According to the foregoing, deformation mode has a strong effect on the microstructure. Bridgman [8] first discovered exceptional microstructure refinement through simple shear induced by HPT. In contrast, the works of Langford and Cohen [44] and other authors on SPD by wire drawing detected fibrous microstructures with substructures of cells and sub-grains but not new grains. Such drawing was performed in numerous passes through slowly convergent dies with insignificant friction when the deformation mode approximates pure shear. Similar lamellar microstructures were also reported for rolling with large reduction and near-pure-shear mode. In contrast, Hirsch et al. [36] observed extremely fine nanograins for rolling in macro-shear bands where deformation mode was changed from pure shear to simple shear. A few attempts have been undertaken to suppress recovery, accumulate dislocations, and develop strain-induced UFG structures by rolling at cryogenic temperatures. However, Wang et al. [45] did not reveal any refinement in copper cryogenically rolled to strain of ε = 5 despite very high density of dislocations, and only after ε > 5 new grains with high-angle boundaries started to appear due to continuous dynamic recrystallization by fragment rotation.

Angella et al. [41] presented a direct comparison of structure refinement by simple shear and pure shear. ECAE of silver produced an ultrafine-grained microstructure with sharp high-angle boundaries and dislocation free-interiors. The material strength increased after the first pass and then showed a plateau. After rolling of the same material with equivalent strains, the structure was formed by sub-grains and cells having diffused boundaries and a high density of dislocations, resulting in a continuous increase of the material strength. Several papers have also been published that compared various SPD techniques regarding structure refinement. In all cases, they confirm the same general trend: the more closely the deformation mode approximates to the simple shear, the more effective the SPD technique is for grain refinement. Therefore, simple shear can be classified as the optimal mode for microstructure refinement and other structure modifications during SPD.

## 5. Principles of SPD Processing

### 5.1. Mechanics of SPD

For uniform processing by pure shear or simple shear, the stress-strain states shown in Figure 1b,c for the small elements should be extended to the whole material with identical conditions at tool boundaries. Kinematics boundary conditions are satisfied if a tool provides rhombus-like material distortion for pure shear (Figure 1b) or parallelogram-like distortion for simple shear (Figure 1c) together with the plane (two-dimensional), uniform stress-strain rate state during finite straining and a fulfillment of the boundary conditions for shear stresses τ developed by contact friction. In the original position, the maximum friction τ = k in the directions shown in Figure 1a requires counter movement of the adjacent tool parts, which is not possible. It becomes even more complicated after distortion ψ (Figure 1b,c) because friction τ along distorted boundaries should be changed in accordance with transformation [28]:

τ = kcos2ψ(10)

Therefore, a uniform simple shear within the bulk rectangular areas oriented in the directions of the principal shears α and β is not achievable.

**Figure 8:**
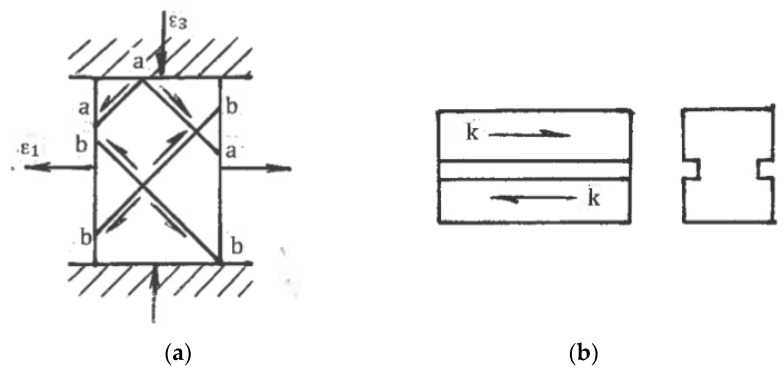
Transition from pure shear to simple shear after flow localization during frictionless upsetting (**a**) and simple shear of thin layer (**b**).

As it was noted in Section 4.1, practical ways to provide pure shear are “ideal” forming operations or multi-pass rolling and drawing with low-contact friction. Frictionless upsetting in the directions of the principal strains ε_1_ and ε_3_ also provides pure shear (Figure 8a) for hardening materials without localization. During SPD with an exhausted hardening ability or even softening, the extensive free surfaces of the upset material present numerous options for localization along a multitude of systems of kinematical admissible shear planes. Figure 8a shows two competitive systems of flow localization “a-a-a” and “b-b-b-b” due to the fluctuation of shear stress. After localization begins, the deformation mode along the acting shear bands is simple shear rather than pure shear. This effect of inversion of the deformation mode explains the inconsistency in some of the results when upsetting was used during SPD to simulate pure shear during rolling. As for simple shear, Rauch and G’Sell [46] demonstrated that a good approximation can be achieved by providing a shear of a thin volume confined between massive material parts (Figure 8b).

**Figure 9:**
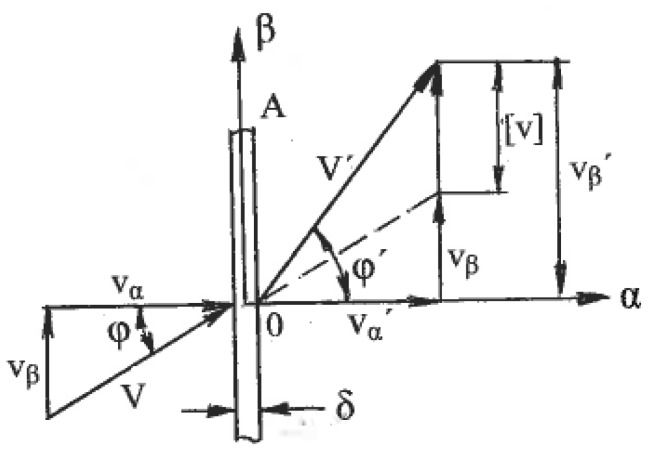
Simple shear along line of velocity discontinuity.

On the other hand, simple shear induced by internal flow localization presents significant practical interest for the processing of bulk materials. For large strains, such localization can be developed naturally or artificially. In the first case, the mechanics of plastic deformation shows strong inhomogeneity in distributions of strains and strain rates within plastic zones and along their boundaries. The general characteristics of most forming operations are thin extended areas of large gradients of strains and strain rates [28,47]. For ideal-plastic solids, these areas are mathematically identified as surfaces of velocity discontinuities along slip lines, which are experimentally observed in most technological operations. When crossing the line A-O (Figure 9) with a continuous normal velocity v_n_ and discontinuity [v] of a tangential velocity, the material particles experience an abrupt change in the flow direction from φ to φ′ and undergo simple shear:

γ = [v]/v_n_ = sin(φ′ − φ)/cosφ′cosφ (11)

**Figure 10:**
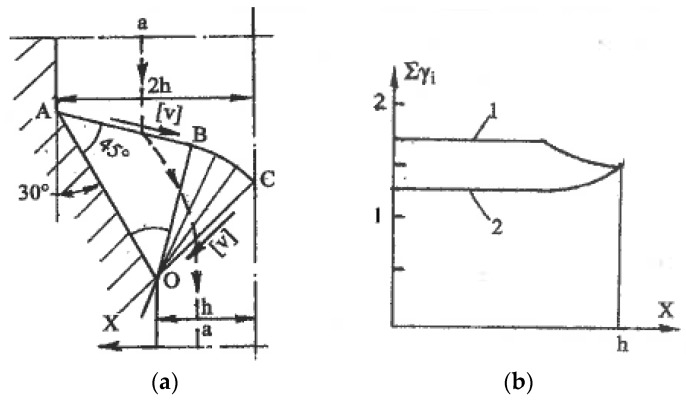
Simple shear processing during extrusion: (**a**) slip line field for extrusion through smooth 30° die with an area reduction of 50%; (**b**) distribution of accumulated shear (1) and shears along lines of velocity discontinuity (2) through section h.

As an example, Figure 10 shows a slip line field (a) and distribution (b) through cross-section h of total shear strain (curve 1) and shear strain (curve 2) accumulated after crossing lines ABC and OC of velocity discontinuities for plane extrusion without friction with an area reduction value of 50% and a die angle of 30° [29]. Sufficiently uniform simple shear comprises the main part of the total shear strains, confirming the potential of using ordinary forming operations for SPD processing.

In the case of artificial localization, the processing mechanics is intentionally designed so that the plastic zone is transformed into a straight thin area of localized simple shear. This can be achieved for specific tool geometry, friction conditions, and kinematics. As a rule, simple shear processing is not accompanied by overall distortions, preserving the material shape and dimensions. Under the careful control of boundary conditions, it also provides strain uniformity with minimum plastic work and load, which are similar to those of “ideal” forming operations. Simple shear techniques will be considered in the next sections.

**Figure 11:**
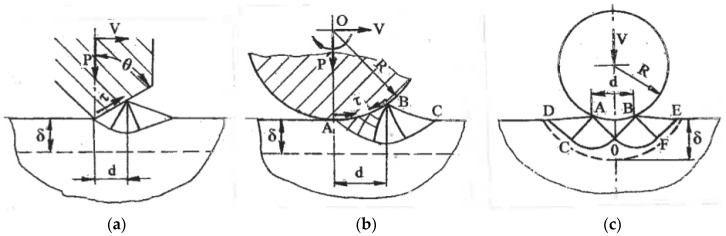
Slip line solutions for surface SPD: (**a**) contact sliding; (**b**) contact rolling; (**c**) contact penetration.

Some applications (wear and corrosion resistance, fatigue) require structure modification in a surface layer. Surface SPD achieved by local plastic contact during sliding, rolling, or impression was considered in [29]. Figure 11 shows slip line solutions for a steady flow during surface sliding (a) and rolling (b) with the formation of a plastic wave in front of the contact area with a tool. The strain rates within the plastic zones are low, whereas shear strains during the crossing of rigid-plastic boundaries with velocity discontinuities are γ ~ 1. Therefore, significant simple shear in the surface layer is accumulated after a few passes of surface sliding or rolling. The slip line solution during impression by a roll (Figure 11c) is unsteady and depends on the d/R ratio. Typically, d/R << 1 and shear strain along a rigid-plastic boundary DCOFE on an order of magnitude is γ ~ d/√2R << 1. Therefore, numerous impressions are necessary to accumulate large shears. The depth of the shear zone in all cases is comparable to the contact length d.

**Figure 12:**
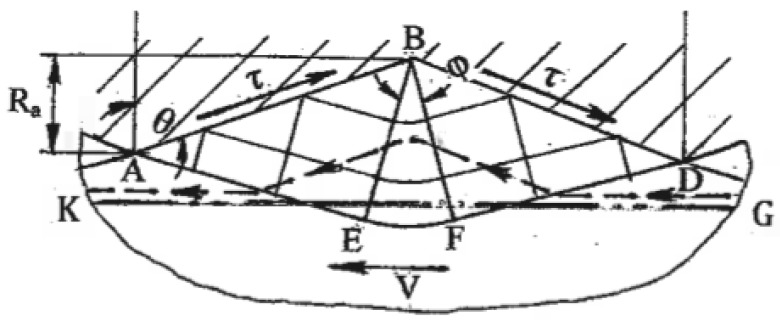
Slip line solution around an asperity during friction SPD.

Another option produces surface shear by contact friction. A plastic state in the subsurface area can be developed under the maximum friction comprising high adhesion and mechanical components induced by interaction with tool asperities. A related model [48] of a steady plastic flow around a unit micro-asperity of the tool surface is shown in Figure 12. Similar to surface sliding and rolling, the shear strain in the subsurface layer is γ ~ 1. During sliding along a plurality of asperities, large shears are developed within the surface layer, where the thickness is comparable to the tool micro roughness R_a_. In some cases of thin materials, surface shear can penetrate through the whole thickness for a distance significantly greater than R_a_.

In the following section, the main focus is on the basic techniques of SPD for the processing of bulk materials with an emphasis on the deformation mode and their practical applications. Obviously, a large variety of other techniques can be realized by a combination of these techniques and with ordinary forming operations.

### 5.2. SPD Technology

Most effects of SPD are observed after strains of ε > 4 at temperatures below the recrystallization temperature. As single-step operations usually cannot develop such large strains, multi-step processing with strain accumulation is necessary. There are also limitations of strain per processing step in conjunction with the strain rate and temperature. For γ > 2, the adiabatic increase of temperature is significant, and processing should be performed at low strain rates to dissipate heat and eliminate overheating, especially at warm processing temperatures. Another challenge is the fabrication of bulk materials with large cross-section areas after SPD. Techniques of simple shear, surface SPD, and friction shear provide a natural way to preserve a material’s shape and dimensions. In contrast, SPD techniques based on ordinary forming operations are associated with essential changes of the cross-section areas. In such cases, cyclic loading or more complex deformation paths are used for periodical restoration of the original shape and dimensions. In terms of the processing mechanics, the effects of SPD also depend on tool geometry and contact friction. For most techniques, friction should be as low as possible. On the contrary, for friction shear techniques, maximum contact friction is necessary. In these cases, significant technical problems are a poor surface finish, intensive tool wear, and high energetic loses induced by friction. Finally, engineering developments of processing tools and equipment have played a key role in transforming SPD techniques into industrial processes and technologies. Though SPD techniques are highly specialized metalworking processes, the general requirements remain the application of ordinary forming equipment; a reasonable cost; and productive, safe, and effective operations including material handling, lubrication, insertion, ejection, and inspection at each processing step. Many new SPD techniques use extrusion “billet-by-billet” production, in which a billet is ejected from a tool by the subsequent billets. Such processing does not allow tool lubrication and inspection after each pass, resulting in a high pressure, material galling of tools, a poor surface finish, and a short tool life. In addition, it is unsuitable for industrial applications because of problems with either ejection of the “last billet” or this billet being left in the tool during inevitable interruptions of processing for short or long durations.

## 6. Processes of SPD

### 6.1. Simple Shear Induced by Friction

Maximum friction requires high normal pressure and clean contact surfaces providing filling of tool micro-asperities and large adhesion shear stress τ (Figure 12). In materials with work-hardening, the plastic flow around micro asperities does not lead to the lowest plastic work. Depending on the boundary conditions, flow localization along other kinematical admissible slip lines, such as line KG in Figure 12, becomes more preferable. Thus, friction shear is transmitted into sub-surface layers, allowing the processing of thin materials. Several processes of friction shear have been developed and applied as SPD techniques.

#### 6.1.1. High-Pressure Torsion

The most important technique is high-pressure torsion (HPT), which was introduced by Bridgman in 1937 [8]. Pippan and Hohenwarten [49] recently published a comprehensive overview of the instrumental aspects of HPT. The exceptional characteristic of this technique is practically unlimited one-step shear, which can be applied to strong and brittle materials under high hydrostatic pressure (up to 8 GPa) at low temperatures, producing the finest microstructures attainable by SPD.

**Figure 13:**
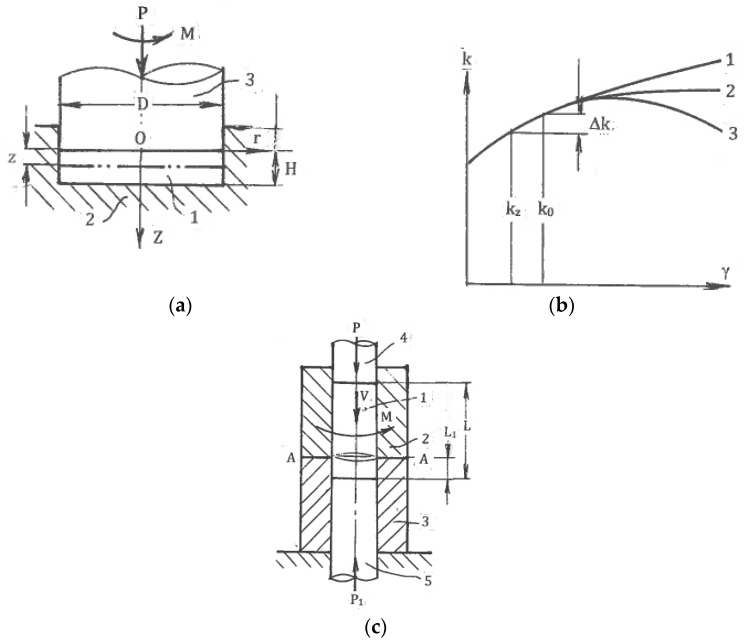
Schematic of high-pressure torsion (HPT): (**a**) fully constrained HPT; (**b**) hardening diagrams; (**c**) HPT of long samples.

However, HPT allows the processing of only small and thin discs. Additional disadvantages are the significant inhomogeneity of strains in the radial and axial directions due to twist straining and shear propagation through the material thickness. This can be explained by the simple model of Figure 13a fully constrained HTP. Sample 1 of diameter D and thickness H is inserted into a die 2. Punch 3 first applies a large compressive pressure P, developing tight contact and large friction at the sample surfaces along with a torsion moment M. Shear is initiated at the contact surface with the punch. After sufficient hardening in the subsurface layer, plastic straining is transferred to lower layers, forming parallel shear zones. Geist et al. [50] clearly observed these zones during shear localization. As pressure P acts nearly hydrostatically, the balance of moments for a material volume confined between sections with axial coordinates z and z = 0 (Figure 13a) gives:

πD^3^k_o_/16 = πD^3^k_z_/16 + πD^2^z(k_o_ + k_z_)/4 (12)

Here, k_o_ is the average shear stress in section z = 0, and k_z_ is the average shear stress in section z. Using the hardening equation k_z_ = k(γ_z_) as a function of the average shear γ_z_ in each layer z with the material hardening coefficient n = dk/dγ (Figure 13b), and assuming that the difference Δγ = (γ_0_ − γ_z_) is small, the last equation gives:

Δγ ≈ 2k_o_z/nD (13)

For SPD, the coefficient n may be positive, zero, or negative (curves 1, 2, and 3 in Figure 13b, respectively). As the largest difference ∆γ takes place at z = H, Equation (13) shows that sufficiently uniform HPT is attained for hardening materials when n > 0 and D/H >> 1. For ideal plastic materials in which n = 0, the difference ∆γ→∞, and shear is localized at the punch surface. For n < 0, k_z_ < k_0_, the difference ∆γ < 0, and shear can be localized in a layer z. All of the considered situations are possible depending on the material and the structure induced by SPD. Therefore, careful control of the processing characteristics is necessary to ensure structure uniformity. This conclusion is confirmed by numerous experimental and analytical results [49,50,51,52].

The industrial potential of HPT is still unclear. The process involves high demands in terms of pressure, torque, and energy, and it is hard to scale up. Additional drawbacks are a very slow processing speed, as well as short tool life and poor surface finish due to intensive dry friction. However, the situation may be changed for very specific applications. Up to now, HPT has remained an effective tool in academic research on SPD in the materials science field.

#### 6.1.2. High-Pressure Torsion of Long Samples

Hohenwarten [53] and Ivanisenko et al. [54] suggested the HPT processing of long billets using an incremental or continuous material transfer through the torsion zone. For the latter case, a simplified scheme is shown in Figure 13c. A cylindrical sample 1 is placed into a split die having a rotating part 2 and a stationary part 3. Punches 4 and 5 squeeze the sample to develop large contact friction and then move it through the die with speed V. Simultaneously, rotating part 2 and punch 4 twists the material across a splitting plane A-A. The concept was experimentally tested for sufficiently short samples and soft materials. The main concerns for practical applications are high contact friction; wasted billet length L_1_, which is necessary to start and complete processing; possible total length L; material ejection from the die; process stability; and complexity of the tool and equipment.

#### 6.1.3. Other Friction Shear Techniques

**Figure 14:**
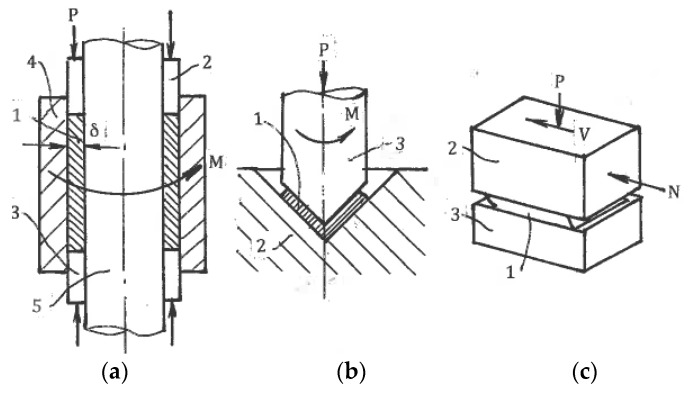
Friction SPD: (**a**) High-pressure tube twisting; (**b**) Cone-cone torsion; (**c**) High-pressure sliding.

Several modifications were also introduced to expand HPT to more complex shapes with a small thickness but large dimensions in other directions:

(a) Toth et al. [55] introduced *high-pressure tube twisting (HPTT)* (Figure 14a) in which sample 1 is compressed by punches 2 and 3 between die 4 and mandrel 5 and is then twisted by rotating die 4. Maximum friction at contact surfaces acting in opposite directions develops shear through a small material thickness δ.

(b) In a *cone-cone (C-C)* method (Figure 14b) developed by Bouaziz et al. [56], sample 1 is compressed and twisted between die 2 and punch 3.

(c) Similarly, Fujioka and Horita [57] described *high-pressure sliding (HPS)* (Figure 14c) for SPD processing of sheet materials. Sample 1 is compressed between anvils 2 and 3 and is sheared by the movement of anvil 2 in forward and backward directions.

These techniques are at the early stage of conceptual proof. As in the case of HPT, the development of corresponding industrial technologies is a challenging engineering problem.

### 6.2. SPD Processes with Internal Simple Shear

Internal shear along lines of velocity discontinuity was considered in Section 5.1. In the optimal case of stress/strain uniformity, the processing mechanics should provide a steady plastic flow through a single straight shear plane under specific boundary conditions.

#### 6.2.1. Equal-Channel Angular Extrusion (ECAE)

**Figure 15:**
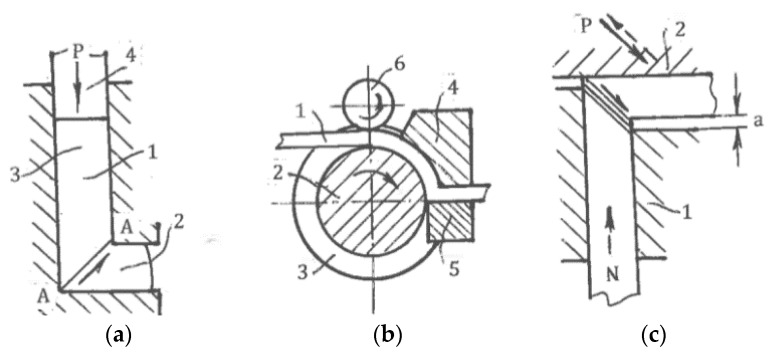
Simple shear SPD: (**a**) Equal-channel angular extrusion/pressing (ECAE/ECAP); (**b**) Continuous ECAE-conform; (**c**) Semi-continuous incremental—ECAP.

ECAE (Figure 15a) was introduced by the author in 1972. The process comprises the extrusion of a material 1 from channel 3 into channel 2 by punch 4 under near frictionless conditions. Thus, intensive, uniform, and strictly oriented simple shear is realized along a crossing plane A-A. A detailed description of ECAE can be found in [29,58]. Presently, this is the most advanced SPD technique and the only one that is used in industrial applications. Ferrasse et al. [59] reported the first ECAE product namely sputtering targets from aluminum and copper alloys fabricated by Honeywell International Inc. Since 2002, the product has remained the only bulk ultrafine-grained material on the market. Therefore, it is reasonable proof of the validity of ECAE technology. Significant improvements were recently made in the adaptation of ECAE to make it suitable for mass production [60]. A new concept of industrial ECAE was successfully tested at Ellwood Texas Forge Inc. The die allows processing the “pass-by-pass” of large plate billets (600 × 600 × 100 mm^3^) without reshaping and reheating between passes, followed by rolling. Thus, relatively inexpensive semi-finished products including plates and sheets with ultrafine-grained structures and superior properties can be produced for various applications. These practical results position ECAE as the cutting-edge SPD technique.

#### 6.2.2. Modifications of ECAE

Many modifications have been proposed to extend the technical capabilities and eliminate the shortcomings of ECAE.

**Figure 16:**
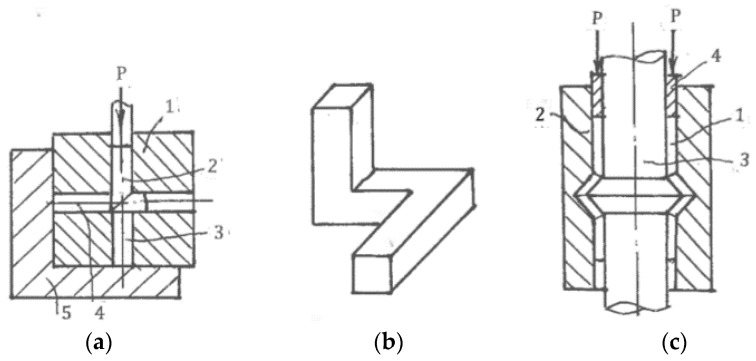
Simple shear SPD: (**a**) Multi-pass ECAE in the rotary die; (**b**) Multi-turn ECAE; (**c**) Tubular channel angular pressing.

(a) Continuous *ECAE-conform* (Figure 15b) for the processing of long materials was first developed in Minsk in 1976 [26]. A long billet 1 is extruded by friction forces through intersecting channels formed by rotating roll 2 with groove 3 and stationary inserts 4 and 5. A coining roll 6 provides a tight contact between the material and roll 2. A similar approach was later used in [61,62,63] and other publications. For thin sheets, ECAE-conform becomes complicated and less productive and economical than rolling of bulk ECAE plate billets. Presently, ECAE-conform is still waiting for effective applications and commercialization.

(b) Rosochowski invented and patented semi-continuous *incremental-ECAP* (I-ECAP) [64]. In comparison with ordinary ECAE, the incremental process (Figure 15c) separates material shearing and feeding for small successive steps “a” that reduces the press capacity and allows the processing of long billets. A few versions of I-ECAP tested on a laboratory scale were presented in [65]. Industrial applications of I-ECAP depend on the development of special and productive tools and equipment.

(c) Nishida et al. [66] introduced *multi-pass ECAE* without billet ejection after each pass in rotary dies or at special presses. Rotary die 1 comprises two crossing channels and additional inserts 3 and 4 overlapping channels, as shown in Figure 16a. Billet 2 is extruded by a punch from a vertical channel into an open horizontal channel. After each stroke, the die is rotated 90°, inserts 3 and 4 change their location in the channels, and processing is repeated. Liu et al. [67] developed another concept of *multi-turn ECAE* in which billets are simultaneously extruded “billet-by-billet” through a few successively located ECAE channels. The two-turn channel is presented in Figure 16b. Both techniques eliminate billet lubrication, reshaping, and reinsertion, but require a complex tool and high pressure, and lead to material galling, a short tool life, and a poor surface finish.

(d) Several attempts have been made to increase strain intensity and to reduce the number of ECAE passes. They include an inlet channel with a variable cross-section, additional twist, and small angular steps [68,69]; a transverse shear and expanding zone between channels [70,71]; and an additional area reduction step in the outlet channel [72]. These and similar modifications significantly increase extrusion pressure, decrease the billet length, and require extrusion “billet-by-billet” without channel lubrication and inspection. They also reduce the efficacy of SPD processing because of deviation from simple shear deformation mode.

(e) Farojiet et al. [73], Mesbah et al. [74], and others developed techniques of *tubular-channel angular pressing (TCAP)*. As an example, Figure 16c shows three-steps TCAP of pipe sample 1 through channels formed between die 2 and mandrel 3. These techniques can only be applied to relatively short tubes. Additionally, significant problems occur in multi-pass processing without lubrication between passes, product ejection from the tool, surface finish, and so forth.

#### 6.2.3. Twist-Extrusion (TE)

**Figure 17:**
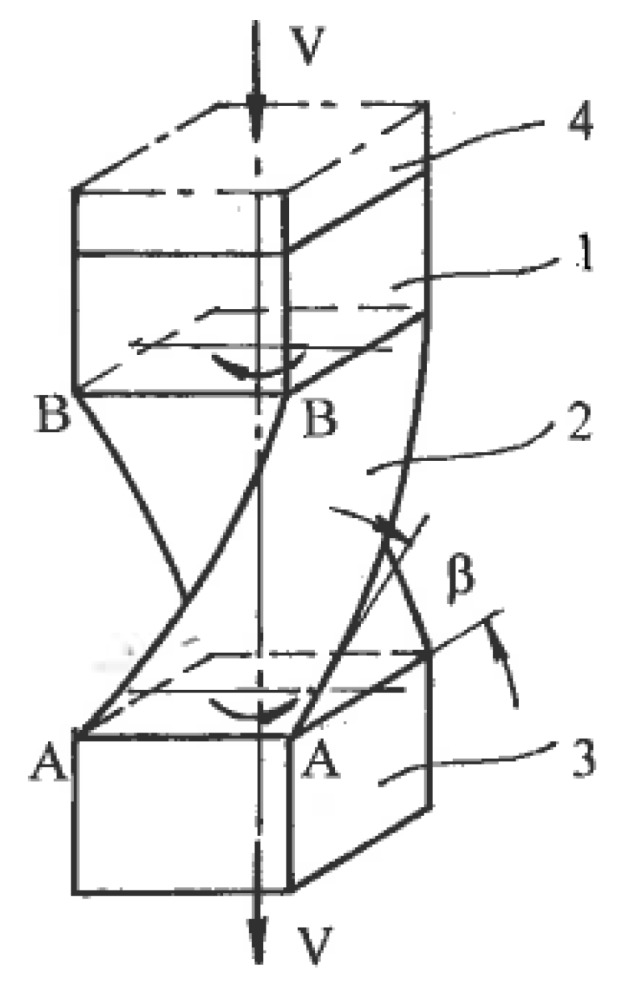
Twist-extrusion (TE).

Beygelzimer et al. [75] introduced the *twist-extrusion (TE)* process (Figure 17), which exploits localized twisting between rigid parts of the material. Rectangular billet 1 is extruded from chamber 4 through spiral die 2 into chamber 3 of the same cross-section areas. During crossing planes A-A and B-B, the material particles experience simple shears by twisting in opposite directions. A detailed description of TE can be found in Beylgezimer et al. [76]. From a practical point of view, problems of TE include strain non-uniformity in a radial direction, tool complexity, contact friction, and the necessity of extrusion “billet-by billet”. Wang et al., 2012 extended twist–extrusion for the processing of cylindrical billets by transforming a round cross-section into an elliptical cross-section, twisting this section into a spiral, and transforming it back into a round cross-section.

### 6.3. Imitation of Simple Shear

**Figure 18:**
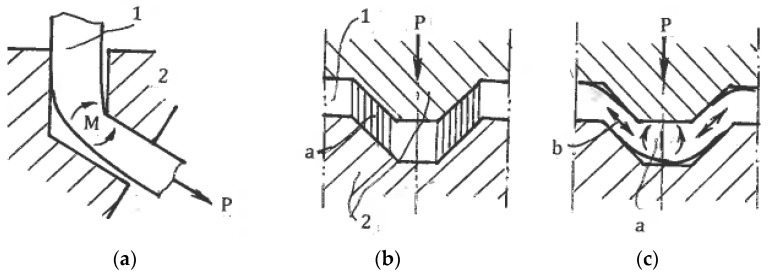
Simple shear imitation: (**a**) Equal-channel angular drawing; (**b**) Supposed shear zones during constrained groove pressing (CGP); (**c**) Actual tensile–bending zones during CGP.

There are also SPD techniques that, to some extent, imitate simple shear. Chakkingal et al. [77] introduced *equal-channel angular drawing (ECAD)* (Figure 18a) as an alternative to ECAE for the processing of long billets and sheet materials. However, tensile load P applied to the exit end of billet 1 changes the processing mechanics from simple shear to tensile bending around a sharp corner with material thinning and near pure shear deformation mode. Alkorta et al. [78] confirmed this conclusion using FEM modeling. It is notable that after multi-pass ECAD, despite significant material hardening, typical characteristics of SPD such as shear bands and new high angle boundaries are not observed.

**Figure 19:**
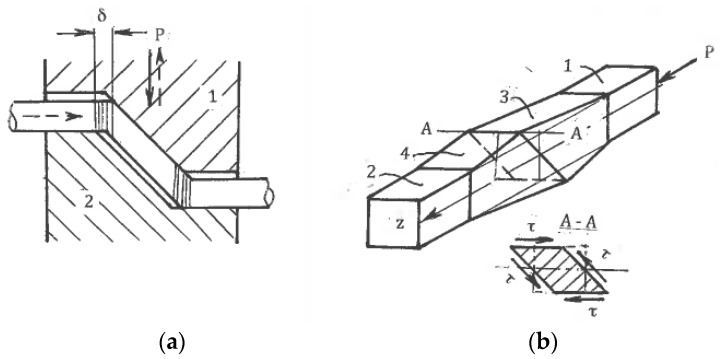
Equal-channel angular swaging (**a**) and “simple shear extrusion” (**b**).

Huang et al. [79] introduced another technique of simple shear imitation—*constrained groove pressing (CGP)* (Figure 18b)*.* Sheet material 1 is periodically formed between tooth-like die 2 and is then straightened between flat dies. It was supposed that areas “a” are subjected to simple shear. However, in reality, the processing mechanics conforms to material bending in areas “a” and stretching in areas “b” during the forming stage (Figure 18c), as well as re-bending and upsetting into related areas during the straightening stage. In all cases, the deformation mode approximates pure shear. In fact, Zrnik et al. [80] showed that structure refinement during multi-pass CGP is sluggish and produces inhomogeneous structures showing a low fraction of high-angle boundaries and new grains. In contrast, Bruder et al. [81] demonstrated that near-simple-shear approximation is observed in identical areas “δ” for *equal-channel angular swaging (ECAS)* due to material constraining by the tool, small incremental feeding steps δ, and the elimination of material re-bending at each step (Figure 19a). This processing is similar to incremental ECAE shown in Figure 15c.

Another example of imitation is the “*simple shear extrusion” (SSE)* induced by Pardis and Ebrahimi [82]. A rectangular sample is extruded with a lubricant by punch pressure P through channels 1 and 2 connected by areas 3 and 4, providing parallelogram-like distortions into the right and opposite directions (Figure 19b). Although the global material distortion geometrically resembles that seen with simple shear (Figure 1c), the actual stress-strain state is three-dimensional and much more complex. In particular, simple shear requires specific shear stresses at the contact surfaces shown in Figure 1c, while the friction τ in Figure 19b (see section A-A) is small and acts in opposite directions. Also, significant shear stresses and corresponding strains between z sections should be taken into account. The related processing mechanics is three-dimensional, highly inhomogeneous, and only approximates simple shear in some small areas, as has been clearly shown in the related finite-element simulations.

### 6.4. SPD by Ordinary Forming Operations

**Figure 20:**
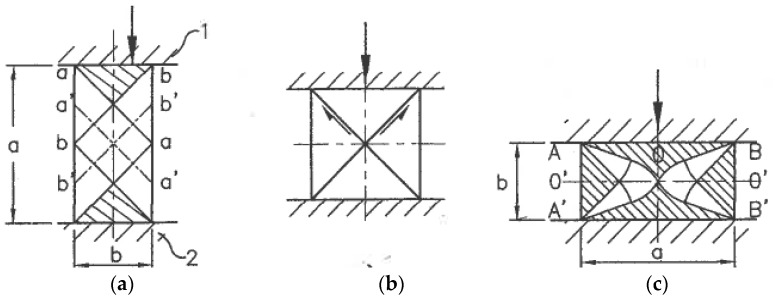
Plastic zones during two-directional plane forging: (**a**) Original position; (**b**) Intermediate position; (**c**) Final position.

*Multi-Directional Forging (MDF)*, the oldest and simplest method of metal forming, was first used for SPD processing by Imaev et al. [83]. It comprises successive steps of upsetting into two (plane) or three mutually perpendicular directions. In the last case, the original material of a rectangular shape A × B × C, A > B > C, is forged along the first axis A until the inverse ratio B × A × C has turned 90°. Then, it is forged along the second axis A until ratio C × B × A has turned 90° again. Finally, it is forged along the third axis A until restoration of the original shape A × B × C occurs. Such non-monotonic, cyclic cross loading is repeated many times to accumulate large strains. Slip-line solutions for three stages of unsteady plane forging (Figure 20) show that intensive simple shear mostly takes place along rigid-plastic boundaries. Salischev et al. [84] showed that these areas are close to the diagonals of the cross-sections, developing significant inhomogeneity of the structure and properties. Important technical advantages of multi-directional forging are its simplicity; low cost; applicability to different materials, including high strength and hard to deform ones, in wide ranges of temperature and strain rate. MDF has been extensively used in academic research and is a prospective candidate for industrial applications.

*Accumulative Roll-Bonding (ARB).* As previously noted, pure shear during rolling does not result in structure refinement, whereas dry friction along a contact area of the material with rolls induces intensive shear and refinement in a thin surface layer (Figure 6b). Saito et al. [85] applied this effect to enhance diffusion bonding and adopt ordinary rolling for SPD of bulk sheets. ARB processing, recently updated by Tsuji [86], includes multi-step rolling with a reduction of 50%, material cutting for two pieces, surface cleaning, stacking, and subsequent rolling with diffusion bonding at each step. This process provides severe straining with just 50% total reduction of the original thickness in the final product. Similar to cryogenic rolling [45], the accumulation of large strains and high density of dislocations during ARB without dynamic recrystallization does not induce new high angle boundaries and structure refinement. Experiments on intensive cold and warm ARB of IF steel carried out by Jamaati et al. [87] and Lee et al. [88] did not find structure refinement, and samples subjected to the process exhibited a laminated microstructure of cellblocks, cells, and sub-grains with low-angle boundaries providing high strength and low ductility. In some cases, the ultrafine-grained microstructure in ARB processed materials can be induced by additional recovery annealing [88]. ARB is labor intensive and time consuming. UFG plate and sheet products rolled from large-scale plates produced by ECAE appear to be more cost effective.

A similar idea of multi-step SPD with material cutting, cleaning, stacking, and bonding at each step was applied to forging [89] and extrusion [90]. Technically, these processing routes are more complex and do not seem as expedient as ARB for sheets or other SPD techniques for bulk samples.

*Forward extrusion*. Lewandowska et al. [91] demonstrated that *hydro-extrusion (HE),* which is similar to the frictionless extrusion shown in Figure 10, significantly refined microstructures after total area reductions more than 100 times. Processing was performed in a few steps with material separation for short parts after each step. Shahbaz et al. [92], Ma et al. [93], and Li et al. [94] suggested different concepts for introducing additional strains during one step extrusion using, respectively, spiral conical dies, rotating conical dies, multi-step dies, and so forth. These dies only provide moderate improvements at the laboratory scale and did not find further developments as techniques of SPD.

**Figure 21:**
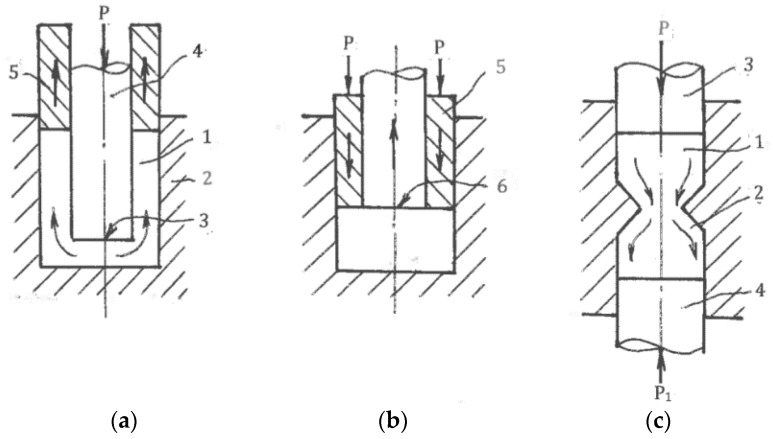
SPD by ordinary forming operations: (**a**) First step of backward extrusion; (**b**) Second step of backward extrusion; (**c**) Cyclic extrusion-compression.

*Backward extrusion.* Fatemi-Varzaneh and Zarei-Hanzani [95] introduced a more promising technique of *accumulative back extrusion (ABE)*. Backward extrusion of material 1 is first performed within container 2 by punch 4 to position 3 (Figure 21a). Then, it is extruded in the opposite direction by ring punch 5 to position 6 (Figure 21b). This processing can be repeated a few times. Characteristics of the related unsteady plastic flow are variable rigid-plastic boundaries with velocity discontinuities causing intensive shear banding and laps [96]. ABE appears especially effective for magnesium alloys owing to structure refinement within shear bands at low processing temperatures and dynamic recrystallization along grain boundaries at hot temperatures [97]. The practical success of ABE depends on the development of industrial tools and equipment, as well as effective lubricants and coatings to prevent surface laps and cracks.

*Combined techniques*. Richert et al. [98] invented *cyclic extrusion-compression (CEC)*—a semi-continuous processing combining extrusion and compression (Figure 21c). For further information, readers are referred to [99]. The main shortcomings of CEC are strain inhomogeneity, which leads to the need for a large number of processing cycles; high pressure; contact friction; and difficulties in scaling up. Among recent developments of combined processes are forward spiral extrusion [100], cyclic expansion-extrusion [101], repetitive extrusion-upsetting [102], and integrated extrusion-ECAP [103]. Although these techniques were demonstrated on a laboratory scale, they present significant technical problems, and their potential for industrial developments and applications is unclear.

### 6.5. Pure Shear SPD: Fiction or Reality?

**Figure 22:**
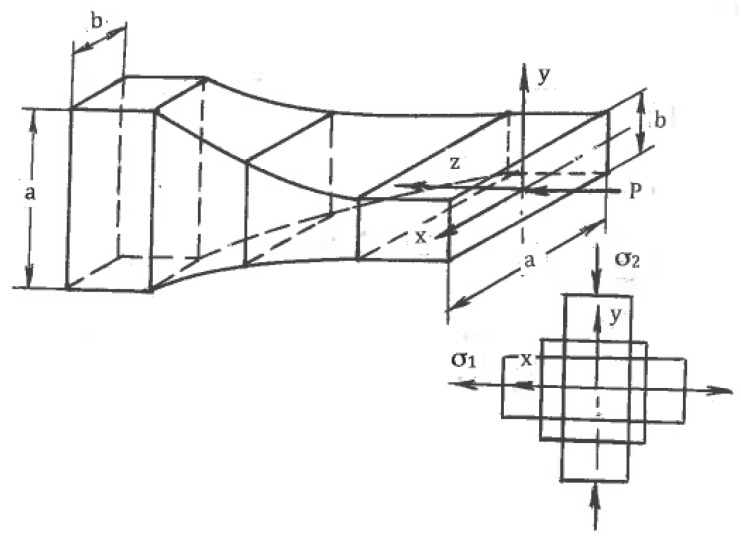
Equal channel forward extrusion.

Mascia and Zhao [104] proposed extrusion through convergent-divergent dies of the constant cross-section area (Figure 22). For rectangular billets, geometrical distortions of cross-sections are similar to those seen when plane pure shear is applied in the directions of the principal stresses σ_1_ and σ_2_, as shown in Figure 1b. Because of this formal similarity, the technique was originally identified as “pure shear extrusion”. However, the actual stress-strain state is more complex because all parameters depend on coordinate z. Later, it was corrected in [105] by acknowledging that the approximation of pure shear only takes place for very small angles of divergence or convergence when gradients in an extrusion direction can be neglected. Otherwise, the flow pattern is three- dimensional and far from pure shear.

**Figure 23:**
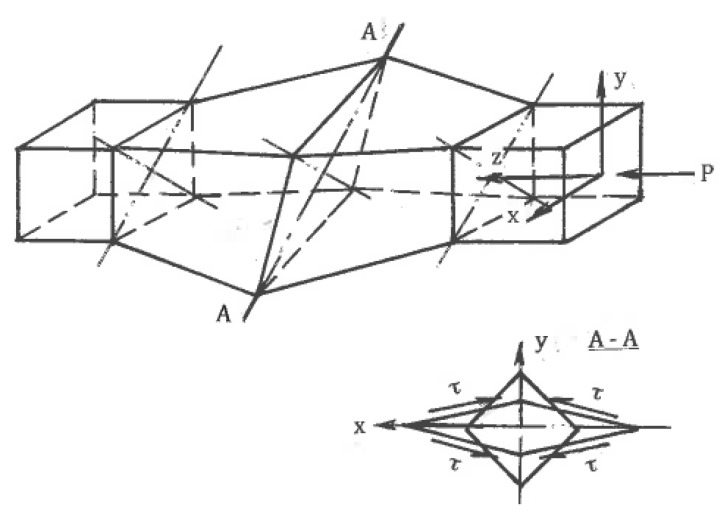
“Pure shear extrusion”.

Recently, Ebrahimi et al. [106] and Rahini and Eivani [107] introduced the same convergent-divergent extrusion as new techniques of SPD. Both approaches used large extrusion angles and effective lubricants. The first approach, *equal-channel forward extrusion (ECFE)*, is identical to that shown in Figure 22 and does not refer to pure shear. The second approach, “*pure shear extrusion” (PSE)* (Figure 23), claimed that *pure shear* is the principal characteristic distinguishing this technique from other SPD techniques. The authors [107] believe that pure shear is provided automatically because global distortions of the material cross-sections (Figure 23) are similar to the rhombus-like distortions of the material elements along slip lines for pure shear (Figure 1b). However, as noted in Section 5.1, additional requirements are plane flow, stress/strain rate uniformity, and surface shear stresses τ in accordance with Equation (10), acting as shown in Figure 1b. In reality, the plastic flow strongly depends on a coordinate z and is highly inhomogeneous, whereas friction stresses (see section A-A in Figure 23) act into opposite directions to Figure 1b. Therefore, pure shear during PSE cannot be achieved. Obviously, the structural effects of SPD with this specific tool design are not induced by a pure shear deformation mode but strong strain non-uniformity, which, like in other extrusion techniques, develops intensive simple shear between different material volumes in the extrusion direction z. PSE uses a more complex tool than ECFE and does not provide additional advantages. Both techniques may have practical potential for some applications, if technical problems of extrusion “billet-by-billet” can be resolved.

## 7. Conclusions

Since the beginning of the intensive research on SPD about 40 years ago, a vast amount of academic activity has generated an avalanche of publications and numerous conferences. In contrast, the technological development of SPD has remained almost at the initial stage. An impartial consideration of the history and literature of SPD research shows that the predominant focus is on microstructural aspects but not processing. Olson [108] noted that the “materials scientist’s distinctive view of structure is defined by the desire to understand the structure and properties relations underlying the technological and economic value of materials … good science—when engineering, manufacturing and economic factors are included in the mix”. With SPD that has not happened, disproportion between fundamental research and engineering practice is obvious, SPD is still far from the age of maturity, and its impact on the industry is insignificant.

The processing mechanics of SPD ranges between pure shear and simple shear. It can be evaluated quantitatively using the coefficient of deformation mode as a scalar and dimensionless characteristic of the strain rate tensor—normalized speed of rotation. Pure shear provides maximum distortion without rotation, delayed localization, the strongest textures, and dislocation hardening, resulting in microstructures of cells and sub-grains with low-angle boundaries. Because of maximum distortion, pure shear is the “ideal” mode for forming operations. On the other hand, simple shear provides minimum distortion, maximum rotation, a strong tendency to flow localization, weak textures, and dislocation hardening. Together with non-monotonic loading, this induces a high density of new high-angle boundaries and structure refinement. The maximum rotation is a signature of simple shear as the optimal mode for structure refinement. The coefficient of deformation mode increases from C = 0 for pure shear to |C| = 1 for simple shear. The quantity of C is a direct index of the efficiency of structure refinement during SPD.

The presented classification of SPD techniques uses simple shear as a baseline. For the effective SPD techniques, deformation mode approximates simple shear with near similar microstructures and properties for identical materials and processing conditions. The considered examples, where pure shear was interpreted as simple shear or simple shear as pure shear, confirmed the rule. However, deformation mode is not the only parameter used for comparison. Very often, technological characteristics are even more important. The required stresses and loads, temperatures and strain rates, tool and equipment, handling operations, lubrication, and so forth determine the cost and practical realization of SPD. These characteristics must be optimized to transform laboratory devices and methodologies into large-scale processes and technologies. Currently, the development of SPD techniques does not go beyond structure characterization and testing of some properties. However, descriptions of laboratory processing and improvements of model materials are not sufficient to demonstrate superior properties. Research activity must be focused on actual problems and advanced alloys. Despite numerous promises and claims, SPD still has not presented effective solutions for topical and specific technical problems. As a result, industry has remained skeptical of its potential.

Analyses of known techniques provide a background for the definition of SPD. *Extensive* hydrostatic pressure is a characteristic of techniques that use simple shear induced by friction. Otherwise, hydrostatic pressures are similar to those of ordinary forming operations or even lower. Processing without any *significant* change of the overall dimensions is associated with simple shear techniques. For techniques based on ordinary forming operations, the overall changes in material dimensions and shape are significant. *Very high* strains are related to SPD techniques with insufficient approximation to simple shear or with large strain non-uniformity. Excessive straining reduces the efficacy of processing and is not justified by the additional increase in performance. For many applications, SPD requires moderate strains rather than severe strains. Similarly, although *exceptional* grain refinement is one of the most intriguing effects, there are many other advantages of using SPD in material science and processing. As this review has shown, simple shear deformation mode is the most universal characteristic of SPD. This conclusion revives the challenging question: what is the principal cause of SPD—large strains, simple shear deformation mode, or both of these?
